# Production and Upgrading of Recovered Carbon Black from the Pyrolysis of End-of-Life Tires

**DOI:** 10.3390/ma15062030

**Published:** 2022-03-09

**Authors:** Sebastião M. R. Costa, David Fowler, Germano A. Carreira, Inês Portugal, Carlos M. Silva

**Affiliations:** 1 Department of Chemistry, CICECO-Aveiro Institute of Materials, University of Aveiro, 3810-193 Aveiro, Portugal; scosta.melo@ua.pt; 2 BB&G-Alternative Worldwide Environmental Solutions, Lda., 2495-402 Fátima, Portugal; david@bbgenv.com (D.F.); germano@bbgenv.com (G.A.C.)

**Keywords:** activation, demineralization, end-of-life tires, pyrolysis, recovered carbon black

## Abstract

Increasing awareness regarding fossil fuel dependence, waste valorization, and greenhouse gas emissions have prompted the emergence of new solutions for numerous markets over the last decades. The tire industry is no exception to this, with a global production of more than 1.5 billion tires per year raising environmental concerns about their end-of-life recycling or disposal. Pyrolysis enables the recovery of both energy and material from end-of-life tires, yielding valuable gas, liquid, and solid fractions. The latter, known as recovered carbon black (rCB), has been extensively researched in the last few years to ensure its quality for market applications. These studies have shown that rCB quality depends on the feedstock composition and pyrolysis conditions such as type of reactor, temperature range, heating rate, and residence time. Recent developments of activation and demineralization techniques target the production of rCB with specific chemical, physical, and morphological properties for singular applications. The automotive industry, which is the highest consumer of carbon black, has set specific targets to incorporate recycled materials (such as rCB) following the principles of sustainability and a circular economy. This review summarizes the pyrolysis of end-of-life tires for the production of syngas, oil, and rCB, focusing on the process conditions and product yield and composition. A further analysis of the characteristics of the solid material is performed, including their influence on the rCB application as a substitute of commercial CB in the tire industry. Purification and modification post-treatment processes for rCB upgrading are also inspected.

## 1. Introduction

The worldwide production of tires is estimated to reach 2.4 billion units per year by 2022 [1]. Tires can be retread and reused (up to a certain point) or disposed of. Due to their nonbiodegradability and bulkiness, landfill disposal of end-of-life tires (ELT) has been gradually replaced by recycling strategies. In Europe, which is accountable for 20% of the manufacture of new tires (300 million passenger car tires and 18 million truck tires, per year [2]), almost 95% of ELTs (3556 Mt) were treated, in 2019, for material recovery (55%) and energy recovery (40%) [3]. This is mostly due to European Union regulations [4] for ELT management that cover not only the collection of tires by particular entities but also their valorization and recycling. Since these policies were adopted, a reduction in tire stock and landfill was observed from 78% (in 1994) to 5% (in 2019) [3].

The tire recovery strategies are highly dependent on their condition, with used tires being classified as scrap tires and part-worn tires. Material recovery from scrap tires includes the following markets: synthetic turf (30%—Europe in 2019), sport and children’s playgrounds (20%), molded objects (26%), asphalt and road paving (2%), and civil engineering (3%) [3]. Regarding energy recovery, scrap tires can be processed in powerplants and co-incineration units due to their heating value (HHV ca. 32 MJ/kg) [5]. On the other hand, part-worn tires may be retreaded (i.e., the tire tread and sidewall rubber are replaced) and reintroduced in the market, contributing to 70% material savings (due to material recovery and extended lifespan) and lower CO_2_ emissions (24%), water consumption (19%), and air pollution by particulate matter (21%) when compared to non-retreadable tires [6]. However, this strategy is not the solution for ELT management because tires are not retreadable forever, and retreaded tires present poorer quality and safety when used at high speed [7].

Even though important steps have been taken for ELT management, research is essential to improve the existing processes and to develop more efficient alternatives. Pyrolysis technologies have a large potential for waste tire valorization since they allow both material and energy recovery within the circular economy concept [8]. In the next sections, this review focuses and systematizes the manufacture of syngas, oil, and recovered carbon black (rCB) as products obtained from ELT pyrolysis. The impact of the process operating conditions on the yield and composition of each product is investigated. Then, a deeper analysis is performed for rCB applications as a substitute for virgin carbon black (CB) in the tire industry and other prospective fields. Lastly, recent studies focusing rCB upgrading for specific applications are presented, including its purification (reduction of organic contamination) and/or modification (activation and ash removal).

**Figure 1:**
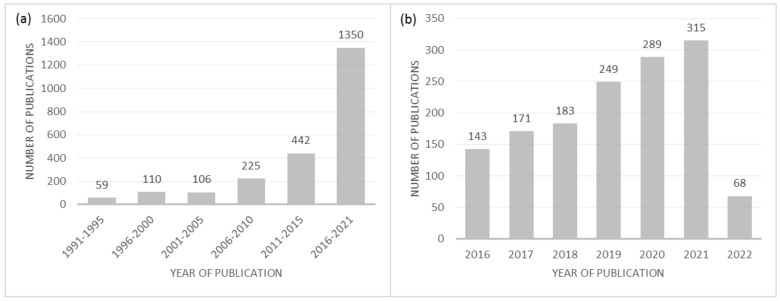
Number of publications on the subject—selected from Web of Science, with the keywords: “pyrolysis” and “end-of-life tires”, “waste tires”, or “used tires”. (**a**) Number of publications between 1991 and 2021; (**b**) Number of publications per year between 2016 and 2022 (March).

A literature search was performed in the Web of Science platform with the following keywords: “end-of-life tires”, “waste tires”, “used tires”, “pyrolysis”, “recovered carbon black”, “carbon activation”, and “carbon demineralization”. The importance of this subject can be inferred by the increasing number of publications over the years. Taking as an example the keywords “pyrolysis” and “end-of-life tires”, “waste tires”, or “used tires”, over 2300 publications have been issued (Figure 1a), of which more than 50% were published in the last 6 years (Figure 1b).

## 2. Pyrolysis of End-of-Life Tires (ELT)

Pyrolysis processes have been used by mankind for centuries to produce charcoal using coal and biomass as feedstock [9]. Pyrolysis consists of the thermal decomposition of organic substances under inert conditions (absence of oxygen) at high temperatures, usually between 400 °C and 800 °C. The inert atmosphere is generally maintained by injection of nitrogen in the system; nevertheless, hydrogen or steam may also be used [10]. Heating the feedstock causes volatilization and decomposition reactions, such as dehydration, cracking, isomerization, dehydrogenation, aromatization, and condensation [11]. Consequently, solid materials are converted into volatile gases and a carbonaceous solid residue (char) that contains the majority of the fixed carbon and inorganics (metals, salts, etc.). After being dragged from the pyrolysis reactor, the volatile gas is separated by condensation/distillation into a condensable fraction (i.e., heavier molecules) and a non-condensable fraction (i.e., low-molecular-weight gases and hydrocarbons, commonly named syngas or pyrogas). Pyrolysis can be coupled to other technologies for further processing of gaseous and liquid products in refineries [12].

When compared to other ELT management strategies, pyrolysis presents several advantages in terms of operational, economic, and environmental aspects. For instance, pyrolysis exhibits higher energetic efficiency and lower emissions of particulate matter and air pollutants (e.g., CO, CO_2_, NO_x_, SO_x_, and polycyclic aromatic hydrocarbons (PAH)) in comparison to incineration [13]. Furthermore, the possibility to integrate other waste materials (such as plastics or biomass) as co-feedstock confers versatility to this technology [14].

The thermal degradation of ELTs is particularly interesting but also challenging, due to their composition, which may include more than 100 components depending on the manufacturing process, trademark, and type of tire [15]. Tires are essentially made of natural and/or synthetic rubber (60–65 wt.%), carbon black (CB) (25–35 wt.%), chemicals and minerals added during the manufacturing process (e.g., vulcanizing agents such as sulfur and zinc oxide and additives such as silicon oxide) [16], and textile, cord, and steel belts used for strength [17]. Martinez et al. [9] summarized the analysis results of various types of tires and reported, on a steel free basis, 57.50–73.74 wt.% volatile content, 19.45–32.28 wt.% fixed carbon, 2.40–20.12 wt.% ash, and 0.40–2.10 wt.% moisture. The dry-base elemental analysis results showed considerable differences for the contents of carbon (67.08–86.70 wt.%) and sulfur (0.92–2.05 wt.%), as well as for hydrogen (6.12–8.10 wt.%) [9]. The differences in feedstock composition pose big challenges for ELT pyrolysis since they impact product consistency.

ELT pyrolysis research has focused essentially on the optimization of pyrolyzer design and operating conditions, as well as their impact on yield, product distribution and composition [9]. There are several types of pyrolysis reactors and several criteria to classify them. For instance, they may be classified regarding the feedstock feed mode (continuous or periodic operation), on the basis of the energy supply mode (heat supplied by the feedstock combustion, by an added inert gas or material, by heat transfer through external walls or internal radiators), or depending on the force used to move the feedstock inside the reactor (i.e., pneumatic, mechanic, or gravitational) [18]. Fixed-bed pyrolysis reactors are the most used for research (at lab scale) despite the disadvantages of its inherent periodic operation (regarding feedstock feed). In this case, the ELT crumb is packed inside the reactor and heated until the set-point temperature, which means it works discontinuously. On the other hand, the continuous mode of operation is considered easier to operate, more flexible concerning the adjustment of operating conditions, and generally more consistent in terms of products composition [19].

The type of reactor and the process operating conditions (temperature, residence time, heating rate, and feedstock particle size) influence the pyrolysis yield, product distribution, and quality [20,21]. Temperature has been identified as the principal variable [9], with higher temperatures promoting syngas production (namely, higher C_1_–C_4_ contents) and decreasing the liquid product fraction, because high temperatures promote further thermal cracking of the liquid organic compounds [22]. It has also been reported that high temperatures lead to a reduction in the C_5_–C_10_ fraction and an increase in the C_10_^+^ aromatic compounds due to Diels–Alder reactions [23].

## 3. ELT Pyrolysis Products

**Figure 2:**
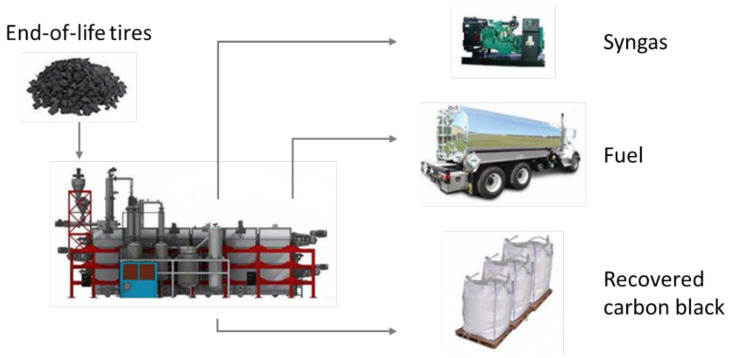
Schematic representation of ELT pyrolysis and ensuing products.

After steel removal, the rubber fraction of ELT enables the production of three distinct pyrolysis products: syngas (gaseous low-molecular-weight compounds), fuel (liquid compounds), and recovered carbon black (rCB), as pictured in Figure 2. The yield and composition of these products are highly dependent on the feedstock composition, the reactor type, and the pyrolysis operating conditions (especially temperature). These features are addressed in the subsections below.

### 3.1. Gaseous Product

The pyrolysis gas product is mainly composed of low-molecular-weight hydrocarbons, namely, C_1_–C_5_ paraffins and olefins [24,25], with traces of sulfur- and nitrogen-containing volatile compounds [26]. A typical composition comprises H_2_ and CH_4_ as main components, with each of these molecules usually accounting for 20–40 vol.% of the total gaseous product [27]. Such pyrolysis gas is frequently called syngas, notwithstanding the conventional composition of a syngas which also includes carbon monoxide in a significant percentage (usually in the range of 20–50 vol.% [28]). The syngas obtained by ELT pyrolysis includes CO and CO_2_ in the range 1–2 vol.% each, other hydrocarbons (frequently C_2_H_4_, C_2_H_6_, C_3_H_6_, C_3_H_8_, C_4_H_6_, C_4_H_8_, C_4_H_10_, and C_5_ and C_6_ molecules), sulfur-containing compounds (usually around 1 vol.%, e.g., H_2_S, SO_2_, mercaptans), and nitrogen-containing compounds (<0.1 vol.% NH_3_) [22,24,25]. Typically, the syngas represents 10–30 wt.% of the pyrolysis products [9].

Syngas production is highly dependent on the pyrolysis operating conditions, as well as on the reactor type and feedstock composition, with higher syngas yields being achieved for higher temperatures and residence times [27,29]. However, long exposure times and high temperatures promote the transformation of saturated hydrocarbons (ethane, propane, and butane) into unsaturated compounds (such as ethylene and propene) [30,31]; hence, the ELT syngas heating value (gross or high heating value (HHV)) becomes lower. Leung et al. [22] studied the influence of ELT pyrolysis conditions (in the range 500–1000 °C) on the syngas HHV and reported it to be within 20–37 MJ/Nm^3^, with the maximum value being achieved in the range 700–800 °C. These results are consistent with other reports in the literature (30–40 MJ/Nm^3^ [32]) and higher than that of wood pyrolysis gas (17 MJ/Nm^3^ [22]). The production of syngas with high HHV is advantageous for pyrolysis systems since it can be directly used in the process making it energy-sufficient [10].

### 3.2. Liquid Product

The liquid resulting from ELT pyrolysis is a brown-colored fuel resembling a petroleum fraction. The pyrolysis yield for the liquid product (34–42 wt.%) is higher than for the other products [33] and is strongly influenced by the technology (type of reactor) and operating conditions (e.g., high pyrolysis temperatures have been reported to reduce the oil yield [34]). In contrast, the chemical composition does not change significantly with the pyrolysis operating conditions [12], although high temperatures have been reported to enhance oil aromaticity [23,31].

Pyrolysis oil is a very complex mixture of compounds (more than 100 molecules have been identified [35]) including alkylated benzenes, naphthalenes, phenanthrenes, *n*-alkanes (C_11_–C_24_), alkenes (C_8_–C_15_), nitrogen- and oxygen-containing compounds in relatively low concentrations, and sulfur [10]. In the majority of cases, the sulfur content (typically in the range 0.55–3.95 wt.%) exceeds the standard limit of 1 wt.%, thus imposing desulfurization of pyrolysis oil prior to its use [33]. In situ sulfur removal techniques include the addition of catalysts or sorbents to the pyrolysis process [36].

The calorific value of ELT pyrolysis oils reaches 44 MJ/kg [9], which is higher than the feedstock’s HHV (ca. 32 MJ/kg [5]) and is close to that of diesel (42–43 MJ/kg) and biodiesel (35.6–44.2 MJ/kg) [33]. Hence, several studies address the combustion of pyrolysis oil for the partial or total replacement of conventional fuel in automobile engines. Encouraging results have been reported in terms of engine performance [37], despite the lower cetane index and the higher density, viscosity, and aromatic content of ELT pyrolysis oils [38]. Nevertheless, the direct use of pyrolysis oil is not recommended due to considerable emissions of NO_x_, CO_x_, SO_x_, and particulate matter [38].

Another area of interest is related to the presence of high-added-value chemicals (such as toluene, xylene, and limonene) in pyrolysis oil. Limonene is particularly interesting since its yield can reach 2.55 wt.% under optimized conditions [39], and it has several applications, for instance, in the production of resins, adhesives, and fragrances [12,40].

### 3.3. Solid Product

The solid product from ELT pyrolysis, commonly known as recovered carbon black (rCB), is a complex mixture arising from the compounds used during tire manufacture, namely, (i) carbon black (CB), (ii) inorganic additives and fillers (such as zinc oxide, silicon oxide, and calcium carbonate), and (iii) traces of steel (recall that metals are removed from the tires prior to pyrolysis) [9]. Repolymerization reactions of rubber compounds may yield additional nonorganized carbon residues [41] estimated to be around 4 wt.% of the total solid product [16]. Hence, the fixed carbon content of rCB is attributed essentially to the CB content of tires.

The feedstock composition, the pyrolysis technology, and the operating conditions impact the yield and characteristics of rCB [9]. Typically, the rCB fraction represents 35–40 wt.% of the total ELT pyrolysis products, thus impacting the economic viability of the process. Hence, researchers have focused on the optimization of pyrolysis conditions for the production of high-quality rCB with commercial application [5,26,42].

## 4. Recovered Carbon Black

### 4.1. Production of rCB by ELT Pyrolysis

**Figure 3:**
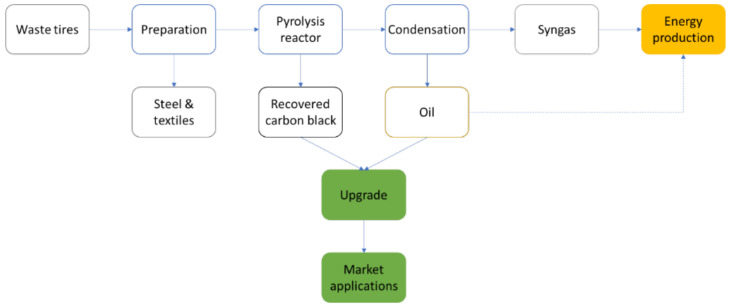
Pyrolysis process: from ELT to rCB, oil, and syngas, and applications thereof.

The yield and composition of rCB depend mainly on the feedstock (ELT) composition and the pyrolysis operating conditions, as compiled in Table 1. Over the last few years, numerous investigations have been conducted to optimize rCB production at both laboratory and pilot scale [5,26,42,43]. This optimization of the process conditions is of major importance to produce a marketable solid material. Nevertheless, upgrading methods may be necessary to enhance the rCB properties depending on the applications requirements, as pictured in Figure 3 and discussed in the subsections below.

Table 1 compiles published data for the yield and composition of rCB obtained from ELT pyrolysis. Kyari et al. [44] pyrolyzed ELT from different brands and countries, using a fixed-bed reactor operated at 500 °C. These authors report that feedstock has no significant influence on rCB yield (37.1–41.7 wt.%) but the gaseous and liquid products show clear dissimilarities in terms of composition (e.g., the aromatic profile of the pyrolysis oil varies with the type of tire). Lopez et al. [31] studied the pyrolysis of two different types of tire (type 1—mainly natural rubber; type 2—50/50 wt.% mixture of natural and synthetic rubbers) in a continuous conical spouted-bed reactor at different temperatures and reported substantial differences for rCB yield and composition (for all tested conditions). In both cases, the rCB yield slightly increased with temperature in the range 425 to 600 °C, attributed to the deposition of aromatic compounds on the rCB surface [31]. In contrast, Rodriguez et al. [5] also reported that rCB yield remains constant (ca. 44 wt.%) for temperatures above 500 °C, and that pyrolysis is incomplete below 500 °C, with the rCB yield being considerably higher (e.g., 87.6 wt.% at 300 °C) due to the presence of a large amount of volatile matter in the solid product. Yazdani et al. [42] reported a similar trend where the rCB yield decreases with increasing temperature, from 43.7 to 21.7 wt.% in the range 400–1050 °C.

Aylón et al. [27] compared the performance of fixed-bed and moving-bed reactors (semi-continuous and continuous operation, respectively) operating at 600 °C, revealing that pyrolysis was complete in both installations with comparable rCB yields (38 wt.%). The rCB samples presented low volatile content (2.51 and 3.50 wt.%) and similar ash content (13.82 and 13.17 wt.%) [27].

Li et al. [45] studied the pyrolysis of scrap tires in a rotary kiln reactor in the range 500–600 °C, showing that temperature has no significant impact on rCB yield (41.3 and 39.3 wt.%, for 500 °C and 600 °C, respectively). However, higher temperatures reduce the rCB volatile content, from 16.14 wt.% (at 500 °C) to 5.86 wt.% (at 600 °C) [45]. Using the same type of reactor, Galvagno et al. [46] obtained higher rCB yields (e.g., 47.4 wt.% at 600 °C) and reported a similar effect of temperature on the volatile content (e.g., 12.78 wt.% at 550 °C and 5.24 wt.% at 680 °C). Moreover, these authors mentioned that high temperatures seem to reduce the H/C ratio, thus indicating a larger aromatization of rCB [46].

Lopez et al. [47] studied the pyrolysis of waste truck tires in a conical spouted-bed reactor, in the range of 425–575 °C. Their results indicate that rubber degradation is incomplete (i.e., lower fixed carbon content) at 425 °C and that rCB volatile content decreases with temperature (from 13.86 to 2.72 wt.%, at 425 and 575 °C, respectively) revealing lower organic contamination within the porous structure [46].

Concerning sulfur content, all rCB samples present high values, ranging between 1.22 and 3.63 wt.%. Aylon et al. [27] reported that the rCB sulfur represents around 64 wt.% of the feedstock’s sulfur content, and its presence is related to the formation of metal sulfides during the pyrolysis process.

In addition to CB and inorganic compounds used during tire manufacture, rCB contains other carbonaceous residues (3–4 wt.% [5]), namely, amorphous deposits (similar to coke) formed by secondary reactions involving the polymer-derived compounds. The extent of these reactions is a consequence of the severity of the pyrolysis process [48]. The repolymerization is based on dealkylation and dehydrogenation reactions of organic vapors contained in the pyrolysis reactor or organic compounds adsorbed on the rCB surface [26]. The carbonaceous residue has a disordered structure [50], and its size is approximately that of five condensed rings [51].

In general, the presence of carbonaceous residues influences the chemical and morphological properties of rCB; thus, it is important to understand the conditions that promote their formation. Pantea et al. [41] performed pyrolysis experiments at atmospheric pressure (100 kPa) and under vacuum (10 kPa) and reported lower contents of carbonaceous residues for the latter conditions. This occurs because, under vacuum, the concentration of gaseous hydrocarbons is lower and, therefore, the phenomena responsible for the residue formation (i.e., adsorption of organic molecules on the rCB surface and the reactions between the volatile compounds) are hindered. Overall, low pressure [41] and high temperatures [52] are recommended to avoid the deposition of carbonaceous residues on rCB, thus yielding a product with chemical and morphological properties comparable to those of commercial CB [51,52].

### 4.2. Commercial Applications for rCB

The production of rCB is focused on the partial or total substitution of CB produced from fossil fuels. Accordingly, it is critical to optimize the pyrolysis process and/or implement further processing stages so that rCB achieves chemical and morphological properties analogous to CB [53]. In this section, the challenges involving the commercial CB replacement by rCB are discussed, evidencing the impact of rCB properties on its application. Moreover, a description of rCB upgrading processes (purification and modification studies) is provided for enhancing the material reinforcing properties for its application in the tire industry.

#### 4.2.1. Carbon Black

Carbon black (CB) is solid carbon produced by combustion or thermal decomposition of fossil fuels (gaseous or liquid hydrocarbons, such as natural gas, petroleum, and coal oil) in highly controlled processes [54]. The process conditions are optimized to obtain specific characteristics which determine the market grades of CB [55]. The identification of CB grades following the ASTM protocol [56] uses a letter and three digits (e.g., NYxx where N stands for a normal curing of the CB, Y is a number based on the particle surface area, and xx is arbitrary). The ASTM nomenclature for CB grades is presented in Table 2, and typical CB grades and properties are presented in Table 3.

CB is a versatile material with many different applications in several industries. It is mainly used as reinforcing filler (ca. 90%) in the production of rubber products, especially tires [57,58], but also as a pigment (for black, bluish, brown, and grey colors) or as a conductive filler for paints and printing inks [59,60], coatings, fibers, polymers [61,62], and batteries [63]. Moreover, it can be used as a UV absorption agent in the polymer industry and as an additive for adjusting the viscosity of inks and paints [57,58].

The CB application area depends on its properties, and these are defined in the early stages of production. The key factors for CB application in certain areas are dispersibility, conductivity, tinting strength, UV stabilization, or abrasion resistance [64]. Table 3 presents typical properties of several grades of CB used for tire manufacture [56,65].

#### 4.2.2. rCB Applications

**Figure 4:**
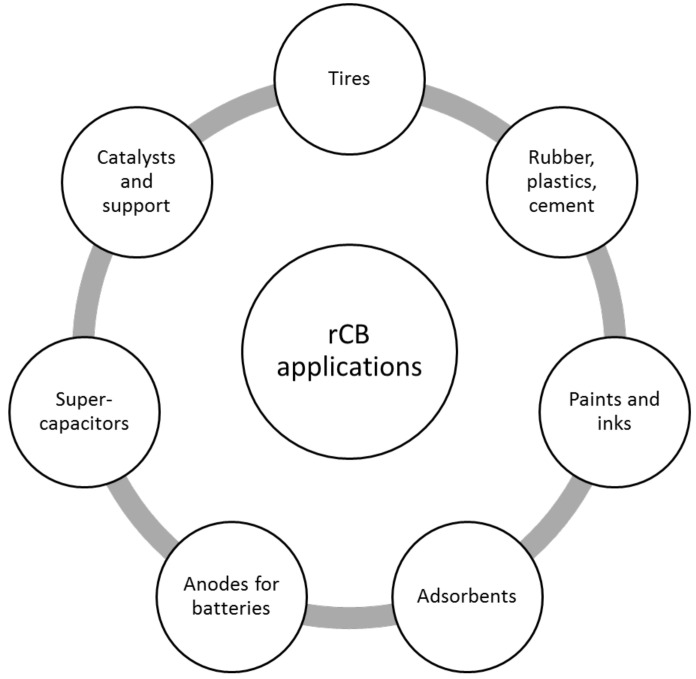
Prospective application fields of rCB.

Due to its chemical and morphological properties, rCB can be used as, for example, filler (for rubbers, bitumen and plastics) or pigment (for the ink industry) [66,67]. Moreover, after further processing [68,69], these activated carbon materials can be employed in a large variety of fields, as depicted in Figure 4. In recent studies, activated rCB samples were investigated as adsorbents for the separation of compounds in gaseous [70,71,72] and liquid [73] phases (e.g., adsorption of dyes [74,75], organic compounds [76,77,78,79], and heavy metals [80,81,82]), as conductive additives for carbon electrodes of sodium and lithium batteries [83,84,85,86], as supercapacitors [87], as catalysts [88,89] and as nanomaterial precursors [90,91]. Even though rCB has been identified as a potential solid fuel (the HHV is within 25 to 34 MJ/kg [9,21]), its low reactivity, slow oxidation kinetics, small particle size, and low bulk density explain why rCB combustion studies are rarely found.

Since the production of rCB is mainly focused on the partial (or total) substitution of conventional CB, the chemical and morphological properties of rCB should be analogous to those of CB. However, substantial differences are perceived between both materials, compromising the direct application of rCB [45]. For instance, Huang et al. [52] observed an irregular distribution of rCB particle size, evidencing that it corresponds to a blend of CB grades used in distinct parts of the tires. Recalling that rCB is mainly obtained from the CB contained in tires, it is obvious that the chemical and morphological properties of rCB will never be those of a specific CB series being closer to an average value of the properties of the CB grades that compose the pyrolyzed ELT granulate [66]. Another difference between rCB and CB is the ash content, which corresponds to 10–20 wt.% in rCB (depending on the feedstock composition) and is below 0.5 wt.% in CB (this is the maximum ash content admissible for tire manufacture) [92]. Another drawback of rCB is the deposition of carbonaceous residues which are responsible for the blockage or deactivation of the active sites [35]. Ash and carbon deposits hinder rCB–polymer interactions, preventing rCB applications in rubber matrixes.

ELT pyrolysis studies frequently characterize the rCB total surface area (nitrogen surface area, NSA, or Brunauer–Emmett–Teller surface area, BET) and structure porosity since these parameters are essential for CB grading. Mikulova et al. [50] reported that increasing the pyrolysis temperature from 450 to 600 °C increases the surface area (from 41 to 88 m^2^/g) and total pore volume (from 0.466 to 0.691 cm^3^/g) of rCB and confers microporosity to the solid. Lopez et al. [47] reported a similar influence of pyrolysis temperature (in the range 425–575 °C) on the BET surface area (it goes from 45.6 to 80.5 m^2^/g) and total pore volume (it increases from 0.215 to 0.337 cm^3^/g). The positive effect of high temperature on these parameters is related to the greater loss of high-molecular-weight hydrocarbons and the promotion of pore opening and widening [47]. Lopez et al. [31] also reported the positive impact of temperature (in the range 425–600 °C) on BET surface areas and the strong relation between this parameter and the feedstock composition. In fact, the rCB BET surface areas were within 61.1–84.1 m^2^/g for a natural rubber feedstock and in the range 36.9–116.3 m^2^/g for a 50/50 wt.% mixture of natural and synthetic rubbers [31]. Li et al. [45] reported a BET surface area of 89.1 m^2^/g and a total pore volume of 0.053 cm^3^/g for rCB produced at 550 °C in a continuous rotary kiln reactor pilot unit. Moreover, N_2_ adsorption and mercury intrusion methods revealed low microporosity and high mesoporosity [45]. In general, the studies reported the production of rCB with BET surface areas comparable to those of CB, particularly the N330 series (see Table 3).

Recent studies assessed the properties and performance of tire rubbers produced with rCB in partial or total replacement of commercial CB [43,93,94,95,96]. Xu et al. [93] studied the performance of a rubber product comprising rCB (produced at 420 °C and 95–100 kPa) as a substitute of CB (N234 series) revealing that the product meets the quality requirements for low- and medium-quality recycled rubbers when rCB consists of small amounts (20 to 50%) of the filler incorporated in the matrix. Sagar et al. [94] investigated the impact of partial and total substitution of CB (N550 series, BET surface area 47.4 m^2^/g; VM 1.9 wt.%; FC 97.1 wt.%; A 1.0 wt.%) by rCB (BET surface area 46.1 m^2^/g; VM 6.5 wt.%; FC 72.9 wt.%; A 20.6 wt.%) and concluded that the rCB high ash content (20.6 wt.%) has a negative impact on the rubber curing process, crosslink density, and mechanical properties, regardless of the substitution ratio (30%, 50%, 70%, and 100% of the total filler amount). The authors indicated that rCB lacks the ability to substitute the N550 commercial CB due to the presence of impurities, along with the higher ash content [94]. The difficulty in using rCB as a reinforcing agent in rubber formulations was also reported by Urrego-Yepes et al. [95]. These authors revealed different properties when rCB or CB are used in the rubber formulations using filler loads of 20, 30, 40, and 50 phr (parts per hundred rubber). However, in the cases of partial substitution of CB (50% replacement), similar rheological, thermal, structural, and mechanical properties were achieved when the total filler amount was increased, i.e., higher loads of rCB–CB mixtures compensated for the lower reinforcing properties of rCB [95]. Berki et al. [96] studied the properties of styrene–butadiene rubbers containing CB (N330 grade) and rCB with similar surface areas, alone or mixed in proportions of 9:1 and 1:1, respectively, using filler loads of 30, 45, and 60 phr. The results showed the rCB is less dispersible and presents a weaker performance as reinforcing filler, which is attributed to the secondary structure of the material, i.e., rCB has a tendency to form agglomerates (filler-filler interactions) leading to lower rubber–filler interactions [96]. Sharma et al. [43] studied the impact on the rheological and physical properties of rubber matrices when performing a total substitution of CB N774 by rCB. The authors observed that the rCB reinforcing characteristics are similar to those of CB N774, suggesting the feasibility of its application in rubber formulations [43]. Therefore, at present, it seems that rCB does not meet the specifications to be used as high-quality reinforcing filler in polymer matrices, i.e., in tire rubber [97].

The rCB heterogeneity can be attributed to the instability of the pyrolysis process, but above all it is related to the variability of the feedstock (ELT) composition, especially the blend of conventional CB grades used to manufacture the tires [66]. Moreover, despite the high carbon content of rCB (FC > 80 wt.%) the dissimilarities of the other parameters (e.g., ash content, particle size, porous structure, surface chemistry, and activity) impart a lack of consistency to the recycled material, limiting its real application and, consequently, compromising the economic feasibility of the ELT pyrolysis process [9]. Ongoing research focuses on the purification and modification of rCB materials in order to make them apt to replace (totally or partially) conventional CB [67].

### 4.3. rCB Purification and Modification

Additional processing methods have been under investigation in order to obtain rCB with reinforcement properties suitable for tire manufacturing. These methods can target the removal of carbonaceous residues (activation), ash content reduction (demineralization), and the reduction in organic volatile contamination [98].

#### 4.3.1. rCB Activation

The activation of rCB may be divided into two categories: physical and chemical activation.

Physical activation consists of a high-temperature (700–1000 °C) treatment in the presence of an activation agent, typically steam or CO_2_ [99]. The rCB pore size distribution and surface area enhancement depend on the activation agent, temperature, and treatment time [35,100]. For example, Choi et al. [101] treated rCB with CO_2_, at 950 °C and with distinct activation periods (1, 2, and 3 h), reporting that total surface area (182–437 m^2^/g) and total porous volume (0.2862–1.3342 cm^3^/g) both increase with time. Furthermore, activated rCB predominantly presented mesopores. González et al. [102] performed activation experiments with steam or CO_2_, in the range 750–900 °C, for 1–3 h, and they concluded that steam promotes higher BET surface areas (85–1317 m^2^/g) when compared to CO_2_ (107–496 m^2^/g) and a narrower microporous profile. Furthermore, increasing both temperature and time leads to higher surface areas of the activated rCB [102]. Cunliffe et al. [103] reported that CO_2_ activation stabilizes the rCB structure (i.e., lower burn-off), but the BET surface areas are 20% lower than those for steam-activated rCB. Activated rCB has been tested as an adsorbent for chromium (VI) [104], butane [105], air pollutants [106], and acid dyes [107].

Chemical activation requires a lower activation temperature and treatment time than physical activation [108,109] since it uses catalysts, such as alkali metals (e.g., KOH, K_2_CO_3_, NaOH, Na_2_CO_3_), alkali-earth metals (e.g., MgCl_2_), and acids (e.g., H_3_PO_4_) [110,111]. Another advantage of chemical activation is the possibility to perform pyrolysis and activation in a single process, i.e., catalytic pyrolysis [110]. Chemical activation influences the mesoporosity and the adsorption capacity of rCB [112]. For example, Acosta et al. [113] reported that KOH-activated rCB presents a high BET surface area (up to 700 m^2^/g) and mesoporosity upon activation in the range 600–800 °C during 1 h. Gupta et al. [114] studied the adsorption capacity of H_2_O_2_-activated rCB (BET area 562 m^2^/g, porous volume 0.97 cm^3^/g) in comparison with a microporous commercial activated carbon (BET area 1168 m^2^/g, porous volume 0.68 cm^3^/g, microporosity 70.59%), for the removal of metal ions (lead and nickel) from simulated wastewaters. The activated rCB exhibited higher adsorption capacity (ca. 96% and 87% removal of lead and nickel, respectively) due to its mesoporous structure (mesoporosity 71.13%, microporosity 28.87%) [114].

##### 4.3.2. rCB Demineralization

One of the main differences between conventional and recovered CB is the ash content, which is below 0.5 wt.% for CB [92] and in the range 10–20 wt.% for rCB [9]. The high ash content has a negative impact on the reinforcing properties of rCB [115], hindering its use for specialized and high-end applications. As mentioned earlier, the rCB inorganic fraction depends mostly on the composition of ELT. Therefore, numerous studies have been conducted to reduce the ash content, usually through an acid–base post-treatment named demineralization [98]. Martínez et al. [97] treated rCB in a stirred tank using HCl (4 M), at 60 °C for 1 h, and reported a substantial reduction in the contents of ash (from 15.0 to 4.9 wt.%) and sulfur (from 2.7 to 0.5 wt.%). Morphological characterization of the treated rCB revealed a slight increase in surface area (from 72.4 to 76.3 m^2^/g), a lower structure, and a higher concentration of surface acidic groups when compared to N550 grade CB [97]. The demineralized rCB can be used as reinforcing filler in tire formulations despite the negative impact on some mechanical properties [97]. Suuberg et al. [116] and Ucar et al. [117] reported similar studies for rCB ash reduction using HCl as a reagent. López et al. [118] analyzed several acid and basic treatments for ash reduction and reported the best results when using a two-stage process in series, with HNO_3_ and H_2_SO_4_ or alternatively HNO_3_ and distilled water (the ash content was reduced from 13.8 wt.% to 6.2 wt.% and 5.3 wt.%, respectively). Moreover, the sulfur content was also significantly reduced by the acid treatment (by 63% with HNO_3_/H_2_SO_4_ and by 68% with HNO_3_/H_2_O) [118]. Zhang et al. [119] used a two-step sequential washing (with HCl and HF) coupled with ultrasonic wave treatment and reported that the rCB ash content dropped from 13.98 to 0.24 wt.% (98.33% reduction). A full characterization of the demineralized rCB proved that its properties (composition, adsorption capacities, surface areas, etc.) were similar to those of N326 grade CB. Cardona-Uribe et al. [120] compared the rCB modification using HNO_3_ and HCl and observed that, even though the ash reduction and surface area were similar for all samples, the HNO_3_ treatment allowed the introduction of important acidic functional groups, such as lactones, phenols, ketones, and carboxylic acids.

#### 4.3.3. Organic Volatile Contamination

In addition to the high ash content and surface contamination with carbonaceous residues, rCB from ELT pyrolysis contains considerable amounts of volatile matter. The volatile contamination is associated with the thermal degradation of rubber polymers and the subsequent adsorption of the ensuing organic compounds on the rCB surface [9]. The characterization of the volatile matter is of particular importance, mainly due to the presence of carcinogenic polycyclic aromatic hydrocarbons (PAH) [121]. The European Chemicals Agency (ECHA) identified eight critical compounds (EU-8 list) whose maximum concentration is limited to 1 ppm, in rubber and plastics for human contact, and to 0.5 ppm, in products for children [122]. The EU-8 list includes the PAH presented in Table 4.

ASTM proposes the quantification of PAH compounds by Soxhlet extraction of rCB samples with toluene, followed by gas chromatography coupled to mass spectroscopy (GC–MS) analysis of the ensuing extracts [123]. Cataldo [124] used GC–MS analysis and Fourier-transform infrared (FTIR) spectroscopy to characterize rubber pyrolysis residues and identified 34 different molecules, including PAHs.

Jonker et al. [125] evaluated a variety of solvents for the extraction of PAH from carbon materials. Pure toluene and mixtures of toluene/methanol and toluene/ethanol were identified as the best solvents for PAH removal, whereas dichloromethane was the poorest [125]. Despite the high efficiency of solid–liquid extractions, these methods involve the use of large quantities of organic solvents. Hence, environmentally friendly solvents must be researched for PAH removal.

Steam activation has been reported to reduce the volatile matter of rCB [126]. This happens because the gas penetrates the porous structure of the solid material, reacting with the organic matter and promoting its elimination/removal. This causes an increase in the rCB surface area, pore volume, and adsorption capacity [108].

High-temperature post-treatments (also named carbonization methods) have been suggested to refine rCB produced by ELT pyrolysis. Helleur et al. [127] investigated the impact of temperature (600 °C and 900 °C) on the post-treatment of rCB under N_2_ atmosphere, for 4 h, using a quartz tube reactor. The organic contamination was assessed by toluene discoloration, a quick and simple test that quantifies toluene-soluble residues in rCB [128]. The toluene transmittance values (TTV) indicated considerable contamination of the starting material (TTV = 14.2%) and low contamination after the post-treatment (TTV of 99.5% and 99.8% after refining at 600 °C and 900 °C, respectively). In a similar way, Martínez et al. [97] reported a decrease in the volatile content (from 4.7 to 2.4 wt.%) after thermal post-treatment along with an increase in the ash content (from 13.2 to 15.0 wt.%) and surface area (from 65.7 to 72.4 m^2^/g). The decrease in carbon content (from 88.0 to 83.0 wt.%) and hydrogen content (from 1.5 to 0.5 wt.%) confirmed the removal of the organic contamination [97]. Pantea et al. [41] investigated the impact of temperature (670, 755, and 870 °C) and pressure (1 and 0.1 atm) on the properties of rCB (produced from waste truck tires) after post-treatment. The rCB weight loss increased with the treatment temperature and pressure, being in the range 2.0–4.5 wt.% and 4.0–7.0 wt.% for the treatments at 0.1 and 1 atm, respectively [41]. Thermogravimetric analysis of the untreated rCB showed that mass changes become noticeable at ca. 300 °C, which correspond to desorption of volatile hydrocarbons and removal of oxygen-containing functional groups from the surface and/or pores of the rCB sample. For higher temperatures, carbon–hydrogen fragments are removed [41].

## 5. Conclusions and Outlook

Global warming and climate changes clearly evidence the need for an industrial paradigm shift. The path for sustainability must focus on the reduction in fossil fuel dependence and CO_2_ emissions and embrace the circular economy concepts. The application of recovered materials in the tire manufacturing industry fulfils these goals. For example, Bridgestone’s goals for 2050 are to use only renewable/recycled materials to replace carbon black (CB) in tire production (ca. 1.8 billion tires per year) [129]. Continental plans to include 10% of recycled materials (e.g., rCB N300) in the composition of tires (ca. 190 million tires per year) by 2025 [130].

Pyrolysis enables the production of new valuable chemicals and energy from waste (e.g., end-of-life tires, ELT). However, continuous improvement is required to optimize the yield and properties of the pyrolytic fuel and the recovered carbon black (rCB). The production of rCB materials from ELT, with chemical and morphological properties similar to those of commercial CB, is currently achievable, but its incorporation in tires as reinforcement filler is still a challenge. Additional research is necessary to guarantee whether rCB can totally replace conventional CB grades. This incorporation is expected to reduce the worldwide CB production by around 90%, thus reducing CO_2_ emissions (a production of 5.7 kg CO_2_ eq. per kgCB is estimated in the case of CB produced by the furnace process) [131].

The application of pyrolysis oil in automotive engines is being examined as a tool to reduce fossil fuel consumption. Moreover, the transformation of pyrolysis oil into carbon black is deemed to be a sustainable route for rCB production [132]. Ongoing research evidences similarities between rCB materials produced from pyrolysis oil and typical feedstock (e.g., ELT) in terms of yield, particle size distribution, surface area, and absorption capacity [133].

## Figures and Tables

**Table 1:** rCB yield and composition reported in several ELT pyrolysis studies.

rCB Yield (wt.%)	Reactor ^(1)^	T (°C)	Proximate Analysis ^(2)^ (wt.%)				Elemental Analysis on a Dry Basis (wt.%)				Ref.
			VM	FC	A	M	C	H	N	S	
38	FBR	600	2.51	83.41	13.82	0.26	81.57	0.84	0.33	2.95	[27]
38	AR	600	3.50	82.09	13.17	1.24	82.10	0.97	0.35	3.41	[27]
37.9	FBR	500	-	-	-	-	82.7	0.4	<0.1	2.2	[44]
39.3	RKR	600	5.86	77.93	14.30	1.98	81.00	1.38	0.51	2.53	[45]
39.9	RKR	550	6.92	77.22	14.58	1.28	80.82	1.46	0.53	2.41	
41.3	RKR	500	16.14	69.19	12.32	2.35	82.17	2.28	0.61	2.32	
48.86	RKR	680	5.24	82.98	11.78	1.44	85.16	0.93	0.22	2.57	[46]
47.40	RKR	600	10.75	76.06	13.19	3.01	85.56	1.33	0.28	2.32	
49.09	RKR	550	12.78	71.89	15.33	3.57	85.31	1.77	0.34	2.13	
38.30	CSBR (truck tire)	600	-	-	-	-	87.24	0.73	0.39	3.37	[31]
36.92		500	-	-	-	-	87.36	0.91	0.44	3.29	
35.36		425	-	-	-	-	86.19	1.25	0.45	3.06	
35.81	CSBR (car tire)	600	-	-	-	-	86.57	7.66	0.44	2.13	[31]
34.05		500	-	-	-	-	86.62	1.39	0.75	2.24	
33.91		425	-	-	-	-	86.46	0.7	0.34	3.59	
40.5	AR	550	4.7	79.3	12.4	3.6	84.4	1.3	0.5	2.3	[25]
35.9	CSBR	575	2.72	87.66	9.62	-	84.98	0.83	0.69	3.63	[47]
35.9	CSBR	475	3.17	87.54	9.29	-	85.71	0.86	0.67	3.28	
37.9	CSBR	425	13.86	77.1	9.04	-	83.81	1.99	0.65	2.96	
33.0	FBR	550	1.2	81.3	16.5	1.0	80.1	0.4	0.2	2.8	[24]
38.0	FBR	500	0.67	90.8	8.41	0.09	90.27	0.26	0.16	1.22	[48]
41.3	AR	475	4.0	75.5	18.5	2.0	76.6	1.4	0.3	3.3	[49]

^(1)^ FBR—fixed-bed reactor; AR—auger reactor; RKR—rotary kiln reactor; CSBR—conical spouted-bed reactor; ^(2)^ VM—volatile matter; FC—fixed carbon; A—ash; M—moisture.

**Table 2:** ASTM nomenclature for CB grades [56].

First Digit (ASTM Grade)	Particle Size (nm)	Surface Area (m^2^/g)
0	0–10	>150
1	11–19	121–150
2	20–25	100–120
3	26–30	70–99
4	31–39	50–69
5	40–48	40–49
6	49–60	33–39
7	61–100	21–32
8	101–200	11–20
9	201–500	0–10

**Table 3:** Typical descriptive values, properties, and applications of CB grades. Adapted from [56,65].

Grade	IV (g/kg)	DBPA (mL/100 g)	c-DBPA (mL/100 g)	NSA (m^2^/g)	STSA (m^2^/g)	Properties	Applications
N110	145	113	97	127	115	High reinforcement and abrasion resistance	Special and off-road tires
N220	121	114	98	114	106	High reinforcement and tear strength	Special and off-road tires
N330	82	102	88	76	75	Medium–high reinforcement; high elongation; good tear and fatigue resistance	Tire tread, carcass and sidewall; bicycle tires
N550	43	121	85	40	39	Medium–high reinforcement; high modulus and hardness	Tire inner liners, carcass and sidewall; hoses and tubing
N660	36	90	74	35	34	Medium reinforcement and modulus; good flex and fatigue resistance; low heat build-up	Tire inner liners, carcass and sidewall; sealing rings; cable jackets; hoses and tubing
N762	27	65	59	29	28	Medium reinforcement; high elongation and resilience; low compression set	Mechanical rubber goods (e.g., extruded profiles and moldings); footwear; rubber flooring
N774	29	72	63	30	29	Medium reinforcement; high loading capacity; low hysteresis	Tire inner liners; footwear; belts and hoses
N990	-	38	37	8	8	Low reinforcement; low modulus, hardness, hysteresis, and tensile strength; high elongation and loading capacity	Tire inner liners; wire insulation and jackets; footwear; belts, hoses, gaskets and O-rings

Legend: IV—iodine absorption number; DBPA—dibutyl phthalate absorption; c-DBPA—compressed dibutyl phthalate absorption; NSA—nitrogen surface area; STSA—statistical thickness surface area.

**Table 4:** List of 8 critical compounds (EU-8 list) identified by the European Chemicals Agency (ECHA) [122].

Benzo(a)pyrene	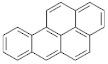	Benzo(b)fluoranthene	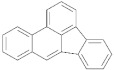
Benzo(e)pyrene	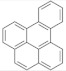	Benzo(j)fluoranthene	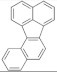
Benz(a)anthracene	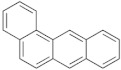	Benzo(k)fluoranthene	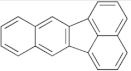
Chrysene	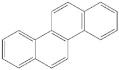	Dibenz(a,h)anthracene	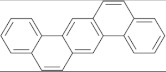

## Data Availability

Not applicable.

## Nomenclature

A: ash, AR: auger reactor, ASTM: American Society for Testing and Materials, BET: Brunauer–Emmett–Teller surface area, CB: carbon black, c-DBPA: compressed dibutyl phthalate absorption, CSBR: conical spouted-bed reactor, DBPA: dibutyl phthalate absorption, ECHA: European Chemicals Agency, ELT: end-of-life tires, EU-8 list: European Union list of eight critical PAH compounds, FBR: fixed-bed reactor, FC: fixed carbon, FTIR: Fourier-transform infrared, GC–MS: gas chromatography coupled to mass spectroscopy, HHV: higher heating value, IV: iodine absorption number, M: moisture, NSA: nitrogen surface area, PAH: polycyclic aromatic hydrocarbons, phr: parts per hundred rubber, rCB: recovered carbon black, RKR: rotary kiln reactor, STSA: statistical thickness surface area, TTV: transmittance value, UV: ultraviolet radiation, VM: volatile matter.
